# Exploring how functional traits modulate species distributions along topographic gradients in Baxian Mountain, North China

**DOI:** 10.1038/s41598-021-04210-x

**Published:** 2022-01-19

**Authors:** Lili Tang, William K. Morris, Mei Zhang, Fuchen Shi, Peter A. Vesk

**Affiliations:** 1 College of Life Sciences, Nankai University, Tianjin, China; 2 School of BioSciences, The University of Melbourne, Melbourne, VIC Australia

**Keywords:** Ecology, Biogeography, Community ecology, Ecological modelling, Forest ecology

## Abstract

The associations between functional traits and species distributions across environments have attracted increasing interest from ecologists and can enhance knowledge about how plants respond to the environments. Here, we applied a hierarchical generalized linear model to quantifying the role of functional traits in plant occurrence across topographic gradients. Functional trait data, including specific leaf area, maximum height, seed mass and stem wood density, together with elevation, aspect and slope, were used in the model. In our results, species responses to elevation and aspect were modulated by maximum height and seed mass. Generally, shorter tree species showed positive responses to incremental elevation, while this trend became negative as the maximum height exceeded 22 m. Most trees with heavy seeds (> 1 mg) preferred more southerly aspects where the soil was drier, and those light-seed trees were opposite. In this study, the roles of maximum height and seed mass in determining species distribution along elevation and aspect gradients were highlighted where plants are confronted with low-temperature and soil moisture deficit conditions. This work contributes to the understanding of how traits may be associated with species occurrence along mesoscale environmental gradients.

## Introduction

Functional traits are associated with environmental conditions and can provide insights into understanding and explaining how plant occurrence changes across different environments. A trait-environment association is a consistent and general pattern linking a biological attribute and an environmental gradient without considering taxonomic identity^1^. Trait-environment associations may mean that only species with particular traits have the opportunity to become abundant under certain environmental conditions. For instance, high-specific leaf area (SLA) species that have fast growth rates and nutrient uptake rates have an advantage in resource-rich environments^2,3^. In contrast, low-SLA species, which have long-lived leaves and a low resource turnover rate, are more tolerant of resource-poor conditions^2,3^.

To identify and measure trait-environment associations, functional traits were incorporated into species distribution models (SDMs)^4–6^, which we call “trait-environment modelling” in this study. The basis of these multilevel models, a coherent hierarchical framework, facilitated a simple interpretation to attempt to describe such associations. In these models, traits are treated as covariates, together with environmental constraints to influence species abundance, and specifically, they are considered mediators between species distribution and the environment^6^.

Plant species distributions are associated with topography at various scales, although the effect is indirect^7,8^. Topography (e.g., elevation, aspect slope) controls microclimate patterns, such as soil moisture and temperature, which in turn influence species distributions^7,8^. For example, soil moisture and microtemperature vary from south-facing slopes to north-facing slopes^9–11^, from high altitudes to low altitudes, and from steep slopes to flat slopes^12^. Many studies have presented the patterns of species response to topography^13–16^. Recently, as functional traits have received more attention, studies have revealed that functional traits are significantly correlated with topography^17^. However, details of how plant traits modulate plant responses to topography are unknown, and understanding the underlying mechanisms could provide knowledge about how topography influences plant distribution.

Here, we applied a trait-environment model to data on species traits and occurrences and topography from a broadleaved deciduous forest in North China to explore the role of functional traits in plant distribution along topographic gradients. We addressed this issue by examining how traits modulate species distributions along topologic changes. To do so, we selected three basic topographic variables (elevation, aspect and slope) and four traits across 31 woody species, including their specific leaf area (SLA), seed mass (SM), stem wood density (SD) and maximum height (MH). These traits represent the leading dimensions of plant ecological strategic variation^3,18,19^ and influence species performance under different environmental conditions^1–3,6^. In addition, to help us understand the trait-topography associations further, we also fitted a model with microclimatic data, while the microclimatic variables we picked here are highly relevant to topology since those topographic variables were our main focus. In this study, to be easily identified, the model with topography is hereby the “topographic model”, and the model with microclimate is the “microclimatic model”.

## Results

### The effects of topography on species prevalence

**Figure 1:**
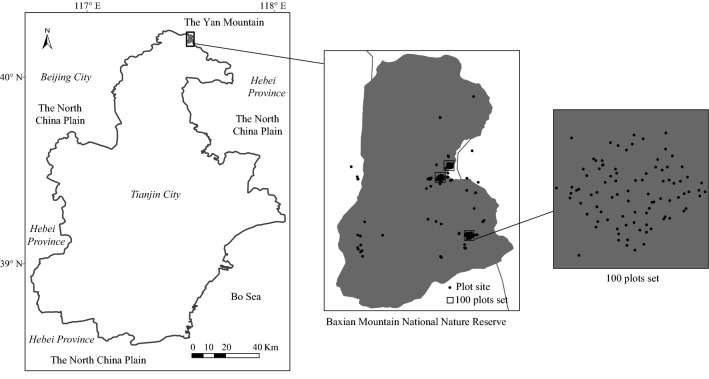
Baxian Mountain National Nature Reserve and plot sites. Three black square boxes in the second map show the location of three plot sets, and each of them has one hundred 10 m × 10 m plots. The red dots in the 100-plot set are plots we sampled and picked. This figure was generated in R language (version 3.6.3).

To avoid adjacent plots introducing spatial autocorrelation, we sampled our investigated plots in the research area (Fig. 1). By repeating this strategy, 10 datasets were yielded to fit the topographic model. According to the Moran’s I results, there are no spatial autocorrelations in the residuals of these 10 models (*P* > 0.05). The coefficient ranges for these 10 fittings are listed in Table 1, and the averaged coefficient results among them can be found in Supplementary Fig. S1 online. Here, we picked the first one to present and discuss the results. The conditional R-squared value of our selected model was 0.55, and the AUROC value across all species was 0.87. According to AUPRC/prevalence, the performance of the topographic model was 4.16 (ranging from 1.17 to 18.90 individually) times better than that of a random classifier.

**Table 1:** Summary of the fixed effects and random effects from our topographic model.

Fixed effect	Coefficient	10 fit ranges	SE	*P* value
Intercept	− 2.23	− 2.22/− 2.31	0.22	< 0.001***
Elevation	0.27	0.22/0.28	0.19	0.14
Slope	− 0.15	− 0.11/− 0.17	0.16	0.34
**Aspect**	0.34	0.28/0.39	0.16	0.03*
SLA: Elevation	0.13	0.12/0.15	0.13	0.31
SLA: Slope	0.07	0.07/0.13	0.09	0.39
SLA: Aspect	− 0.02	0.01/− 0.10	0.09	0.76
SM: Elevation	− 0.14	− 0.14/− 0.19	0.15	0.33
SM: Slope	0.18	0.15/0.23	0.10	0.07
**SM: Aspect**	0.29	0.17/0.31	0.10	0.004**
SD: Elevation	− 0.08	− 0.06/− 0.10	0.15	0.61
SD: Slope	− 0.12	− 0.09/− 0.14	0.10	0.22
SD: Aspect	0.04	0.04/0.10	0.11	0.68
**MH: Elevation**	− 0.32	− 0.25/− 0.33	0.15	0.03*
MH: Slope	− 0.06	− 0.01/− 0.08	0.10	0.58
MH: Aspect	− 0.18	− 0.05/− 0.18	0.11	0.09
Random effect	SD			
Site (Intercept)	1.32			
Species (Intercept)	1.01			
Species (Elevation)	0.70			
Species (Slope)	0.40			
Species (Aspect)	0.42			

*P < 0.05, **P < 0.01, ***P < 0.001.

**Figure 2:**
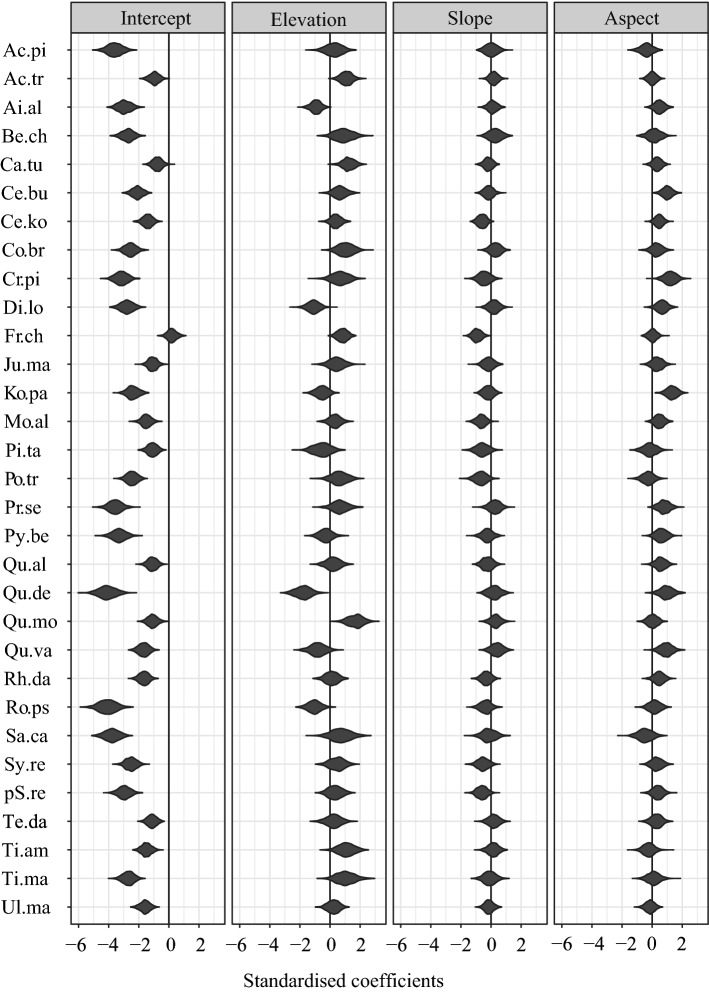
Estimates of fixed effects of environmental variables on 31 species occurrences given their traits. The violins refer to the uncertainty in intercepts and coefficients. Species names were shortened following Supplementary Table S1 online.

The mean prevalence of 31 species on a logit scale was − 2.23 (SE = 0.22) (Table 1), indicating that species with mean trait values have 7% to 11% occurrence under average environmental conditions. The prevalence (intercept) varies widely among species; *Fraxinus chinensis*, *Carpinus turczaninowii*, *Acer truncatum*, *Quercus aliena* and *Q. mongolica* were the most prevalent species (Fig. 2).

Aspect had a greater influence on species occurrence than the other two topographic factors, and its effect was also more consistent across species (Table 1). The results from 10 sample datasets also indicated that (Supplementary Fig. S1 online). In contrast, the effect of elevation on occurrences differed more widely from one species to another (Table 1).

### Trait influence on species response to topography

Species distributions could be modulated by some traits across elevation or aspect gradients (Table 1), not for slopes. Specifically, the maximum height interacting with elevation and seed mass interacting with aspect stood out from our topographic model (Table 1). The results from 10 grid sample datasets also showed this trend, although some coefficients varied across the subsets (Supplementary Fig. S1 online). Specific leaf area and stem wood density were not significantly associated with the variation in plant occurrence along topographic gradients (Table 1, Supplementary Fig. S1 online).

**Figure 3:**
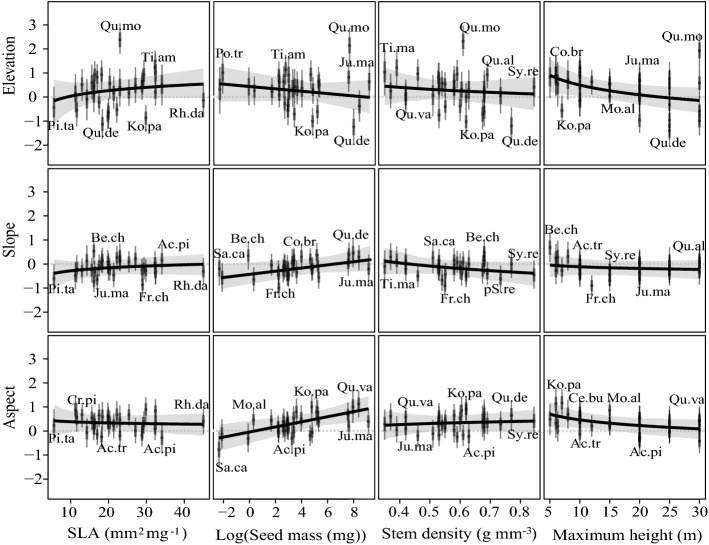
The relationships between environmental responses and species traits given other traits at their medians. The environmental responses were centred, so species with values above zero on the Y-axis had a positive response to the particular environmental variable. Black lines show the coefficient of trait–environment interaction terms in our model. Gray areas refer to the 95% confidence intervals of the estimate. Each boxplot shows the estimated environmental response associated with one particular species trait, given all the other environmental constraints and trait medians. Every boxplot has one square dot with two intervals, representing the mean and 50% and 95% credible intervals. Species names were shortened following Supplementary Table S1 online. For a better illustration, the values of seed mass were log transformed. “*” and “**”indicate that the coefficients of the associations were significant.

Maximum height is the trait that contributes the most to that explanation, with a coefficient of -0.32 and an SE of 0.15 (Table 1). Particularly, the shorter responses to elevation were generally more positive. In contrast, there was an opposite trend when the species reached 22 m and became taller (Fig. 3, top row fourth column). That is, shorter-statured tree species occur more preferentially at higher-altitude sites, while for taller-statured tree species, we could more easily find them at lower-altitude sites.

In addition, the association between seed mass and aspect also had a large and significant coefficient (0.29, SE = 0.10), showing that seed mass modulated the response to aspect more than the other three traits. Furthermore, the association between seed mass and aspect indicated that trees with heavy seeds (> 1 mg) responded more positively to aspect and that light-seed (< 1 mg) trees tended to show negative responses (Fig. 3, the last row, the second column). Thus, the occurrence probability of species with heavier seeds would increase on south-facing sites, while species with lighter seeds were estimated to have the opposite responses to aspect.

## Discussion

We integrated trait-environment associations into GLMM to quantify the modulating ability of traits in species distributions along topographic gradients. Based on the association measurements, we evaluated the strength of the trait effects. Interestingly, in our study, trees with maximum heights greater than 22 m usually had contrary interactions with elevation in comparison with those less than 22 m. Such polarity also occurred between trees with seed masses heavier and lighter than 1 mg in response to aspect gradients. When one trait was close to the particular threshold, accordingly, its mediation became less effective. This threshold definition holds an intrinsic potential to spatialize the vegetation pattern and brings new insight to understand how elevation and aspect shape the species ranges.

### Maximum height modulating species occurrence along an elevation gradient

Our results showed that tree species shorter than 22 m responded positively to elevation, while tall ones (higher than 22 m) showed the opposite response, and such interactions became more obvious when the trees were shorter or taller. Plants' maximum height indicates several ecological strategies they are adopting. First, at low altitudes with abundant resources and minor stress, taller ones are more competitive here and then distribute more because they have a greater chance of getting light than their shorter neighbours^20^. When trees grow taller, due to gravity and path length resistance, increasing leaf water stress may limit leaf expansion and photosynthesis for furth living^21^. Additionally, plants at high elevations face harsher living conditions and prefer to adopt a more resource-conservative growth strategy. In other words, they spend their energy and resources growing into a resistant plant rather than growing tall^22^. Specifically, growing taller than usual can be attained at the cost of plant stem diameter growth and result in less mechanical and physiological support to the crown^22,23^. Moreover, living conditions at high mountains are usually not as friendly to thin stem plants. They are more likely to be broken by strong winds or lighting strikes, but shorter and sturdy individuals are more likely to survive.

The sink limitation hypothesis^24,25^ in treeline formation studies may provide another perspective to understand the general negative relationship. Sink limitation proposes a low-temperature restriction of tissue formation in the uppermost stands. Specifically, cold temperatures at high elevation limit tissue formation in shoots and roots by increasing the concentration of nonstructural carbohydrates (NSCs)^25–28^. In other words, under cold temperature conditions, instead of forming new tissue, more sugars produced in the Calvin cycle reactions are directed into NSCs. Studies showed that root growth was strongly and directly restricted by the soil temperature when it was lower than 6 °C because cell elongation rates would be significantly reduced in that situation^29^. Moreover, shoot meristematic growth is slowed in high elevation stands, probably because of decreasing air temperature^30,31^. To understand this, we collected microenvironmental data, including near-surface extreme cold hours (NSCHs), via microclimate modelling and fit the trait-environment model. The results showed that there was a significantly negative coefficient of the association between maximum height and near-surface extreme cold hours (NSCH) (Supplementary Table S2 online, Supplementary Fig. S3 online).

### Seed mass modulating species distribution along the aspect gradient

According to our results, starting from 1 mg, heavier-seed trees responded positively to southerly slopes and gradually more when the mass value increased, while trees with seed masses lighter than 1 mg showed opposite responses. This can be explained by “seed mass trades off seed number”^32^. Heavy seeds have the advantage of stress tolerance, while light seeds have the advantage of seed yield^3,32^.

Differences in the heat received between south-facing and north-facing slopes will cause the variation of water content in soil^9–11^. Generally, the soil on south-facing sides is drier than that on north-facing sides in the Northern Hemisphere, and this trend was also illustrated in our study area (Supplementary Fig. S2 online). That is, trees on south-facing slopes may face more drought stress in Baxian Mountain. Drier conditions are a challenge to plants with light seeds, while heavier seed trees will be more likely to survive, as they tend to perform better in seedling growth when facing drought and other hazards, most likely because heavier seeds can produce larger seedlings (seedling-size effect)^33,34^. In our study, this trend was indicated by the negative coefficient of the interaction between seed mass and soil moisture (MIO) in the microclimatic data fitted model, although not very certain (Supplementary Table S2 online, Supplementary Fig. S3 online). This result is consistent with most previous results regarding the relationship between seed mass and soil moisture^35–38^.

In addition, plants on north-facing slopes suffer less drought stress. Such less survival stress benefits all local plants, while heavy seed plants produce fewer seeds than light-seed plants, which would lead to a smaller population. As a result, small seed plants flourish more than heavy seed plants on north-facing slopes.

## Research outlook

Trees on the southerly sites are facing more drought stress than the northerlies on Baxian Mountain (Supplementary Fig. S2 online), and we believe it would be interesting if we could introduce one hydraulic trait to our analysis. A previous study showed that drought can lead to xylem cavitation of vascular plants (hydraulic conductivity)^39^, and cavitation will frequently occur when plants obtain too little water so that embolized conduits will no longer be able to hold the sap and the plants will die^40–42^. One easy-to-measure hydraulic trait, xylem vulnerability to embolism (P50)^43^, either to stem or to leaf, could reflect that ability straightforwardly. A study in a tropical rainforest in Brazil showed that species with low P50 (drought-resistant) tend to occur more often in high and well-drained uplands^44^. It is worth considering more in the future to explore its role in species responses to environments.

Previous studies have shown that plants display strong variations in some traits^45,46^, and phenotypic plasticity was found to influence species responses to environments^47–49^. It has also been found that the plasticity among different traits varies. For example, photosynthetic traits were more plastic, while hydraulic and leaf economic traits were less plastic^50^. However, the species in our study covered a large range of families and genera, and in this situation, phylogeny contributed the most trait variations^51^, and such a fitted model could still bring much inspiring results. For future studies, it will be valuable to consider such trait intraspecific variation in our method and explore how plasticity assists plants in responding to changing environmental conditions.

## Methods

### Study area

The species occurrence data, functional traits and environmental variables were collected at the Baxian Mountain National Nature Reserve (40.1836  N, 117.5464 E), Northern China, at elevations between 200 and 1000 m. According to the Köppen climate classification, it is within the hot summer continental climate regime (Dwa)^52^, Figure 1;^53^, Figure 5). The annual average temperature is 12.9 °C (https://web.archive.org/). The warmest month is July, with an average temperature of 26.8 °C, while the coldest month is January, with an average temperature of − 3.4 °C. The annual precipitation is 516 mm. The month with the highest precipitation on average is July, reaching 150 mm, while the lowest month is January, with an average of 3 mm. The reserve is a mostly deciduous broad-leaved forest dominated by *Acer*, *Quercus* and *Juglans* species.

### Species occurrence data collection

We sampled three, one-hectare plot sets along topographic gradients, including 100, 10 m × 10 m plots  (Fig. 1) in each set. Moreover, we broadly located 69, 10 m × 10 m plots outside those three sets along topographic gradients (Fig. 1). To avoid the many plots from the three one-hectare plot sets inducing significant spatial autocorrelation, we resampled from those sets by putting a 3 × 3 grid on each set and randomly picking 3 plots in each grid cell, in the end comprising 150 plots (27 × 3 + 69 = 150) for modelling. Moran’s I was measured to check if the dataset spatial autocreation was successfully limited^54^. We repeated this sampling strategy 10 times, yielding 10 datasets for modelling. In each plot, we recorded the occurrence of every species, and here, we used the data from 31 moderately common woody species. Their scientific names and corresponding abbreviations used in figures are listed in Supplementary Table S1 online.

### Functional trait data collection

We followed the leaf-height-seed (LHS) scheme^19^ and focused on SLA reflecting the fast-slow continuum of leaf economics^55^, MH representing the responses to the balance of productivity and disturbance^3^ and SM reflecting a tolerance-fecundity trade-off^32,56^. In addition, we also selected SD to denote the trade-off between growth and survival in woody plants as the fourth trait dimension^18^.

For 31 woody species, we collected their functional trait data, including SLA (fresh area/dry mass, cm^2^ g^−1^), SD (dry mass/fresh volume, mg mm^−3^), seed mass and maximum height from TRY^57^, China plant trait database^58^, Kew Seed Information database (https://data.kew.org/sid/), Scientific Database of China Plant Species (DCP) (http://db.kib.ac.cn/) and some republished papers^59–61^.

All functional traits ranged widely among the species in this study. Species ranged in SLA from 5.60 to 45.05 m^2^ kg^−1^. The seed mass ranged over nearly four orders of magnitude, from 0.10 to 9259.00 mg. The tallest species had an average height of 30 m, six times that of the shortest (5 m). The stem wood density ranged the least, from 0.35 to 0.85 g cm^−3^.

### Environmental variables

Three topographic variables at the Baxian Mountain Nature Reserve were used: elevation, aspect and slope. We acquired elevation data for each plot from a 30-m resolution digital elevation model (DEM) (Resource and Environment Data Cloud Platform, http://www.resdc.cn/Default.aspx). From this DEM, we derived slope and aspect in ArcGIS (version 10.2) for each plot. For aspect, we transformed the variable to degrees of south-north orientation, from 0° (facing north) to 180° (facing south). Values over 180° were converted by subtracting 360 and multiplying by -1.

### Data preprocessing

Considering that the distributions of our original covariate data were highly skewed, all traits and environmental data except aspect were log-transformed. To interpret the model coefficients more easily, we centred all traits and environmental data and reduced the range by twice the standard deviation^62^. Therefore, intercepts could be explained as overall prevalence given all the mean values of environments and traits, and slope terms could be interpreted as partial dependencies given that other variables have mean values.

### Trait-environment model

The trait-environment model we used was a generalized linear mixed-effect model (GLMM). It was proposed by Pollock et al.^6^ for representing the modulation by traits of the relationship between species occurrence and environmental gradients. We added a site identifier as a random effect to account for nonindependence^5^ and assumed the following:

$$\begin{aligned} & {\text{Logit}}\left( {p_{ij} } \right) = \alpha + a_{j} + \left( {{\varvec{\beta}}_{{1\user2{ }}} + {\varvec{b}}_{{\varvec{j}}} } \right){\text{X}}_{{\varvec{i}}} + {\varvec{\beta}}_{12} {\text{X}}_{{\varvec{i}}} {\text{Z}}_{{\user2{j }}} + c_{i} , \\ & \;i = 1, 2, \ldots , n,\;j = 1,2, \ldots ,m \\ \end{aligned}$$

where $p_{ij}$ is $Pr\left( {y_{ij} = 1} \right)$, referring to the probability of species $j$ occurrence at site $i$. ${\varvec{X}}$ is a matrix of quantitative environmental data for $n$ sites. ${\varvec{Z}}$ is the trait matrix for $m$ species. $\alpha ,{\varvec{\beta}}_{1}$ and ${\varvec{\beta}}_{12}$ are fixed effect terms. $\alpha$ gives the overall prevalence of species across sites given the mean value of each trait for all species and the mean value of each environmental variable for all sites. The vector ${\varvec{\beta}}_{1}$ refers to the average response to each environmental variable given average trait values for all species. The vector ${\varvec{\beta}}_{12}$ has twelve elements (4 traits × 3 environmental variables) and denotes the trait-environment association, indicating how traits modulate species responses to environmental variables. The vector, ${\varvec{b}}_{{\varvec{j}}}$**,** and variables $a_{j}$ and $c_{i}$ are the random effect terms, where ${\varvec{b}}_{{\varvec{j}}}$ describes the response of every species to each environmental variable and $a_{j}$ and $c_{i}$ respectively show the deviations in prevalence at the species and site levels. We did not fully follow the model proposed by Jamil et al.^5^ and recommended by Miller et al.^63^ in our study, functional traits were only incorporated into our model as “trait-environment” interactions rather than fixed effect terms. This means that, rather than directly influence their occurrence, functional traits indirectly influence species occurrence by modulating their response to environments.

We used the *blme*^64^ package to fit our model in a Bayesian setting, which allowed us to specify a particular form of weak prior to obtaining an approximate Bayesian maximum posterior estimation. The prior distribution for the species covariance of random effects was an inverse Wishart distribution with $df = 8$ and a $4 \times 4$ diagonal variance–covariance matrix, and the variance was 2. The prior distribution for the site covariance of random effects was an inverse gamma distribution with shape and scale parameters 0.5 and 100, respectively (default). The prior distribution for fixed effects was a normal distribution with $\mu = 0$ and $sd = 1$. We evaluated model performance by the area under the receiver operating characteristic curve (AUROC) and the area under the precision-recall curve (AUPRC) since the input data of our model are highly skewed^65,66^. AUROC and AUPRC were calculated using the R package *PRROC*^67,68^. In addition, we calculated the values of AUPRC/prevalence for each species, where prevalence here is equal to AUPRC of a random classifier^69^, to show how many times the model’s prediction is better than a random classifier.

We fitted the model with four traits of 31 species and three topographic variables from 150 plots. Based on the 10 datasets from the grid sampling process, we built 10 topographic models and averaged the coefficients for each fixed effect term for visualization (Supplementary Fig. S1 online). Additionally, to help us to understand the associations between topographic variables and functional traits, we fitted this trait-environment model with microclimatic variables predicted by microclimate modelling, which were considered directly associated with plant occurrence underlying the effect of topography. Some results from this model will be mentioned in the discussion section, and the details can be found in the Supplementary methods (see Supplementary methods online for more details).

## Supplementary Information

Supplementary Information.
