# Supplementary material for: Exploring how functional traits modulate species distributions along topographic gradients in Baxian Mountain, North China
Source: Sci Rep. 2022 Jan 19;12:994. doi: 10.1038/s41598-021-04210-x (PMC8770611; doi:10.1038/s41598-021-04210-x)
Supplement: Supplementary file 1 — Supplementary Information. [file 41598_2021_4210_MOESM1_ESM.docx]

**Supplementary information to the paper**

Exploring how functional traits modulate species distributions along topographic gradients in Baxian Mountain, North China

Lili Tang, William K. Morris, Mei Zhang, Fuchen Shi, Peter A. Vesk

Correspondence:

Fuchen Shi, College of Life Sciences, Nankai University, Tianjin, China

Email: fcshi@nankai.edu.com

Phone: + 86 22 23502477

Peter A. Vesk, School of BioSciences, The University of Melbourne, Melbourne, VIC, Australia

Email: pvesk@unimelb.edu.au

Phone: + 61 3 93547480

**Supplementary Table S1.** Scientific names and corresponding abbreviations of 31 species.

|  | Scientific name | Abbreviation |
| --- | --- | --- |
| 1 | *Acer pictum* | Ac.pi |
| 2 | *Acer truncatum* | Ac.tr |
| 3 | *Ailanthus altissima* | Ai.al |
| 4 | *Betula chinensis* | Be.ch |
| 5 | *Carpinus turczaninowii* | Ca.tu |
| 6 | *Celtis bungeana* | Ce.bu |
| 7 | *Celtis koraiensis* | Ce.ko |
| 8 | *Cornus bretschneideri* | Co.br |
| 9 | *Crataegus pinnatifida* var*. major* | Cr.pi |
| 10 | *Diospyros lotus* | Di.lo |
| 11 | *Fraxinus chinensis* | Fr.ch |
| 12 | *Juglans mandshurica* | Ju.ma |
| 13 | *Koelreuteria paniculata* | Ko.pa |
| 14 | *Morus alba* | Mo.al |
| 15 | *Pinus tabuliformis* | Pi.ta |
| 16 | *Populus tremula* var*. davidiana* | Po.tr |
| 17 | *Prunus serrulata* | Pr.se |
| 18 | *Pyrus betulifolia* | Py.be |
| 19 | *Quercus aliena* | Qu.al |
| 20 | *Quercus dentata* | Qu.de |
| 21 | *Quercus mongolica* | Qu.mo |
| 22 | *Quercus variabilis* | Qu.va |
| 23 | *Rhamnus davurica* | Rh.da |
| 24 | *Robinia pseudoacacia* | Ro.ps |
| 25 | *Salix caprea* | Sa.ca |
| 26 | *Syringa reticulata* | Sy.re |
| 27 | *Syringa reticulata* subsp*. pekinensis* | pS.re |
| 28 | *Tetradium daniellii* | Te.da |
| 29 | *Tilia amurensis* | Ti.am |
| 30 | *Tilia mandshurica* | Ti.ma |
| 31 | *Ulmus macrocarpa* | Ul.ma |

**Supplementary Fig. S1. The mean of coefficients of 10 topographic models built by 10 sample datasets**


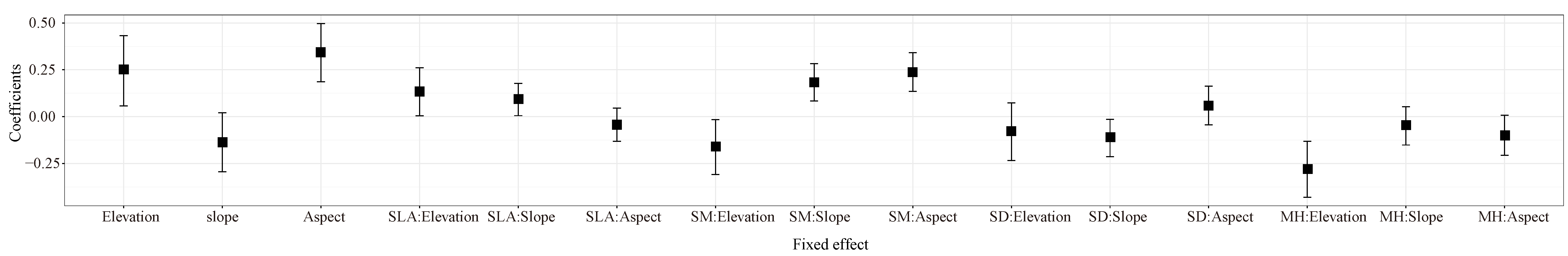


SLA, specific leaf area; SM, seed mass; SD, stem density; MH, maximum height. The figure shows the mean coefficients of fixed effect terms among 10 grid sample datasets. Each coefficient mean has the same trend as its corresponding term in table 1. Error bars are the mean of the SEs from 10 fits.

**Supplementary methods.** Microclimatic model

To understand the associations between topographic features and functional traits, we fitted a model with trait, species occurrence and microclimatic data, where the microclimatic data was considered directly associated with plant occurrence underlying the effect of topography.

All microclimatic data in our study were generated by NicheMapR and microclim models (Kearney, Gillingham, Bramer, Duffy, & Maclean, 2019; Kearney, Isaac, & Porter, 2014; Kearney & Porter, 2017). We calculated out the hourly near-surface temperatures at 5 cm above the ground, hourly soil temperature and annual soil moisture values at 5 cm depth for all 369 plots. We divided the hourly temperature data into growing season (from April to October) and non-growing season (from November to the next March), and respectively set up the thresholds for extremely hot temperature level and extremely cold temperature level. Specifically, values of temperature greater than 90% of the hottest temperature value were considered as extremely hot, and vice versa. Then we counted the hours in those two levels. Overall, the microclimatic data included annual soil moisture (MIO), near-surface extreme cold hours (NSCH), near-surface extreme hot hours (NSHH), soil extreme cold hours (STLH) and soil extreme hot hours (STHH).

A PCA was conducted among all three topographic and five microclimatic features to select uncorrelated microclimatic variables used in our microclimatic model in R language. We picked plots’ soil moisture and near-surface extreme cold hours data according to the PCA result of environmental variables (Supplementary Fig. S2) and fitted a model with them. These microclimatic variables are considered directly associated with plant occurrence underlying the effect of topography. Based on the 10 datasets from grid sampling process, we built up 10 microclimatic models, and averaged the coefficients for each fixed effect terms for visualization.

In our microclimatic model, most interaction coefficients were not influential (Supplementary Table S2, Supplementary Fig. S3). One clear trait-environment interaction was that shorter species showed more positive responses toward more extreme near-surface cold temperatures (Supplementary Table S2, Supplementary Fig. S3). In the future study, we will measure the microclimatic data and fit the model again, these directly measuring data may yielded different results.


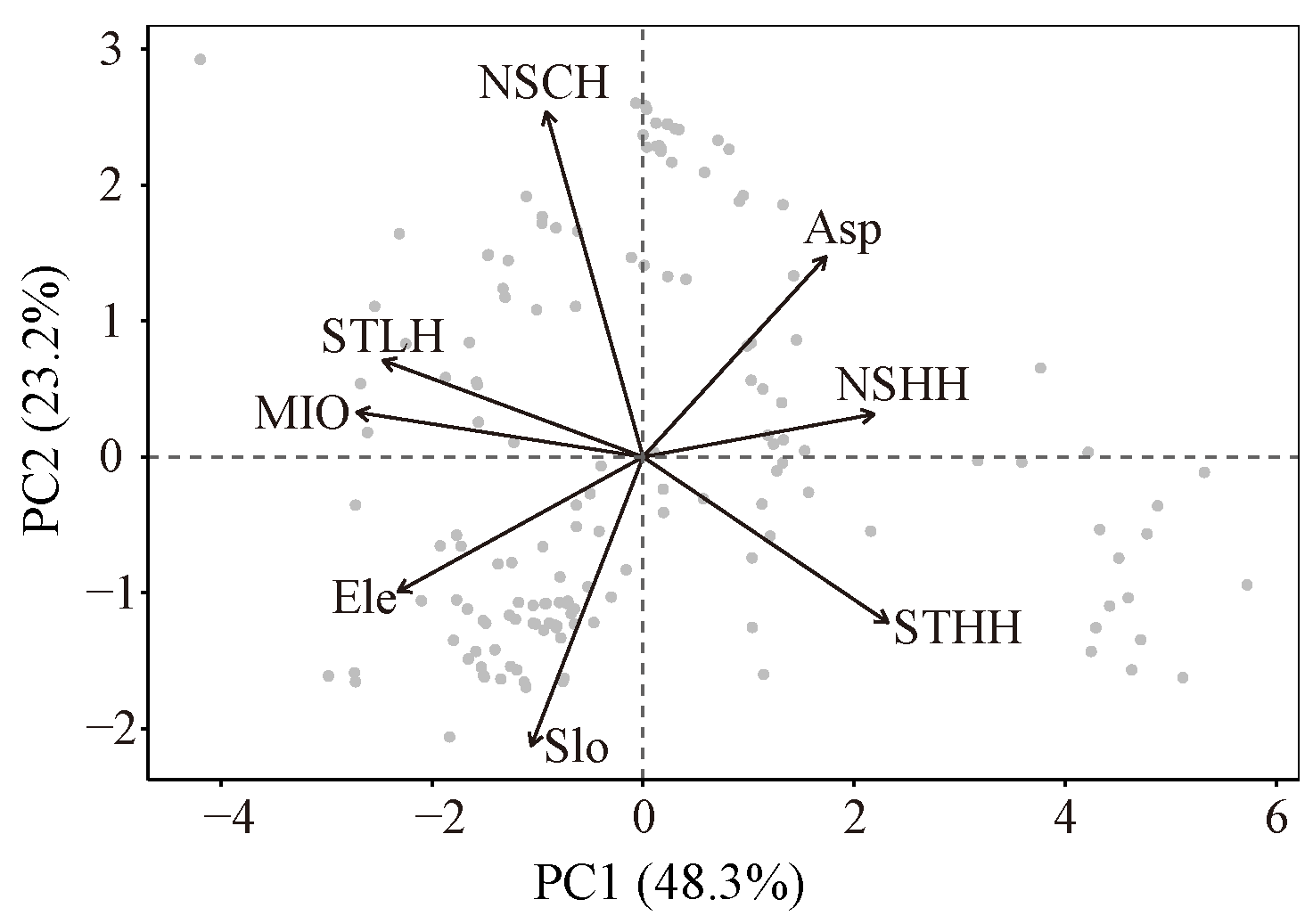


**Supplementary Fig. S2.** The PCA result of environmental variables. Asp, aspect (south); Ele, elevation; MIO, annual soil moisture; NSCH, near-surface extreme cold hours; NSHH, near-surface extreme hot hours; Slo, slope; STLH, soil extreme cold hours; STHH, soil extreme hot hours.

**Supplementary Table S2.** Summary of the fixed effects from our microclimatic model.

| Fixed effect | Coefficient | 10 fit ranges | SE | p-value |
| --- | --- | --- | --- | --- |
| Intercept | -2.04 | -2.03/-2.13 | 0.20 | <0.001 *** |
| MIO | -0.10 | -0.08/-0.19 | 0.13 | 0.42 |
| NSCH | 0.21 | 0.14/0.23 | 0.11 | 0.06 |
| SLA: MIO | 0.13 | 0.10/0.16 | 0.11 | 0.24 |
| SLA: NSCH | -0.03 | -0.08/0.02 | 0.09 | 0.76 |
| SM: MIO | -0.15 | -0.07/-0.16 | 0.13 | 0.25 |
| SM: NSCH | 0.12 | 0.01/0.14 | 0.11 | 0.28 |
| SD: MIO | -0.12 | -0.08/-0.12 | 0.13 | 0.37 |
| SD: NSCH | 0.14 | 0.10/0.15 | 0.11 | 0.18 |
| MH: MIO | -0.14 | -0.14/-0.20 | 0.13 | 0.26 |
| MH: NSCH | -0.22 | -0.17/-0.25 | 0.11 | 0.04* |

MIO, annual soil moisture; NSCH, near-surface extreme cold hours; SLA, specific leaf area; SM, seed mass; SD, stem density; MH, maximum height.


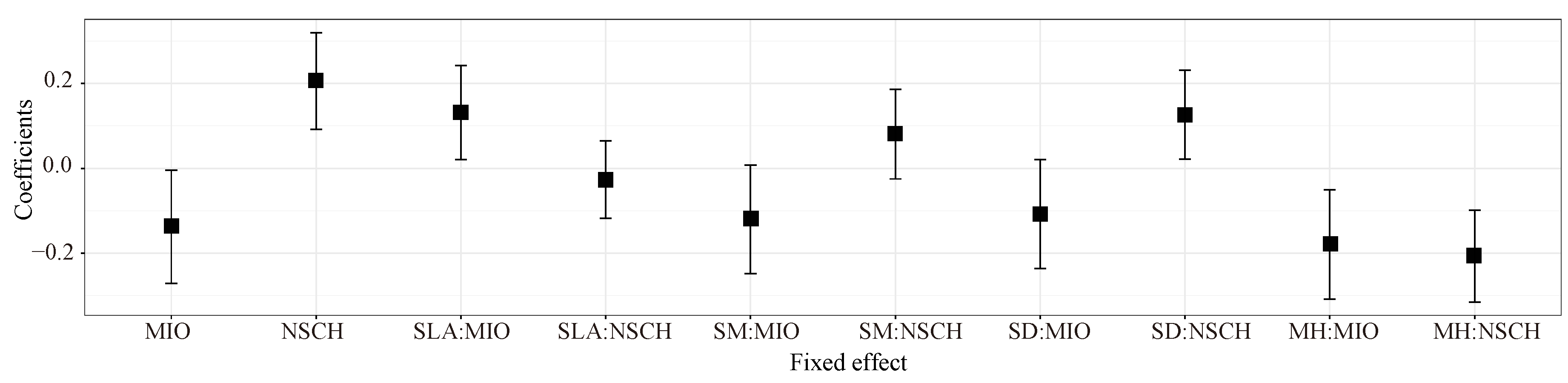


**Supplementary Fig. S3.** The mean of coefficients of 10 microclimatic models built by 10 sample datasets. MIO, annual soil moisture; NSCH, near-surface extreme cold hours; SLA, specific leaf area; SM, seed mass; SD, stem density; MH, maximum height. The figure shows the mean coefficients of fixed effect terms among 10 grid sample datasets. Each coefficient mean has the same trend as its corresponding term in table 1. Error bars are the mean of the SEs from 10 fits.

Reference

Kearney, M. R., Gillingham, P. K., Bramer, I., Duffy, J. P., & Maclean, I. M. D. (2019). A method for computing hourly, historical, terrain-corrected microclimate anywhere on Earth. *Methods in Ecology and Evolution*, n/a(n/a). https://doi.org/10.1111/2041-210X.13330

Kearney, M. R., Isaac, A. P., & Porter, W. P. (2014). microclim: Global estimates of hourly microclimate based on long-term monthly climate averages. *Scientific Data*, 1(1), 140006. https://doi.org/10.1038/sdata.2014.6

Kearney, M. R., & Porter, W. P. (2017). NicheMapR – an R package for biophysical modelling: the microclimate model. Ecography, 40(5), 664–674. https://doi.org/10.1111/ecog.02360
